# CLPTM1L Is Overexpressed in Lung Cancer and Associated with Apoptosis

**DOI:** 10.1371/journal.pone.0052598

**Published:** 2012-12-26

**Authors:** Zhenhua Ni, Kun Tao, Guo Chen, Qingge Chen, Jianmin Tang, Xuming Luo, Peihao Yin, Jihong Tang, Xiongbiao Wang

**Affiliations:** 1 Central Lab, Putuo Hospital, Shanghai University of Traditional Chinese Medicine, Shanghai, China; 2 Department of Pathology, Shanghai Changning Central Hospital, Shanghai, China; University of Saarland Medical School, Germany

## Abstract

CLPTM1L is believed to be associated with lung cancer. However, there is little information regarding its expression and function. Here using immunohistochemistry, we found that CLPTM1L expression was markedly increased in lung cancer tissues relative to normal tissues, especially in lung adenocarcinoma. CLPTM1L expression was not found to be associated with stages, smoking status, lymph node metastasis, or T lymphocyte infiltration but with differentiation stage. We found CLPTM1L to be enriched in the mitochondrial compared with plasma membrane protein extracts. CLPTM1L-EGFP transfection showed that the molecule product was expressed in cytoplasm and indicated the mitochondrial localization stained with mitochondrial marker MitoTracker. CLPTM1L transferred lung cancer cell line 95-D showed no growth inhibition or cell apoptosis, but it did show inhibited sensitivity to cis-diamminedichloroplatinum(II) (cisplatin, CDDP). Knockdown of CLPTM1L by RNAi did not interfere with cell proliferation but it did increase cell sensitivity to CDDP and activation of caspase-9 and caspase-3/7. These data indicate CLPTM1L is a mitochondria protein and that it may be associated with anti-apoptotic mechanism which affects drug-resistance in turn.

## Introduction

Palate transmembrane 1-like (CLPTM1L), also called *c*isplatin *r*esistance *r*elated gene 9 (CRR9), was identified among the genes involved in resistance to the anticancer drug cisplatin in ovarian cancer cells [1]. CLPTM1L is located at the 5p15.33 locus near telomerase reverse transcriptase [TERT]. Recent genetic studies revealed that this locus is a susceptibility region for lung and several other cancers [2], [3], [4], [5], [6], [7], [8], [9], [10], [11], [12], [13], [14], [15], [16]. It seems likely natural that genetic variants would cause abnormal expression at the protein level and that this may influence the gene function in lung cancer. Several studies have shown CLPTM1L to be highly expressed in renal carcinoma cell line and laryngeal squamous cell carcinoma [17], [18].

The fact that CLPTM1L is so highly conserved indicates that it is probably important to some basic function. However, little is known about what CLPTM1L and its product or products actually do. The molecule has, however, been found to be a drug-resistance factor [1]. The expression of CLPTM1L was found to be upregulated in all cisplatin-resistant cell lines examined. The overexpression of CLPTM1L in a cisplatin-sensitive cell line was found to cause apoptosis, and CLPTM1L over-expression was found to have no effect on cisplatin-resistant cells.

Primary lung cancer is the leading cause of cancer deaths in most industrialized countries [19]. The powerful genetic association between CLPTM1L and lung cancer inspired us to address the expression and function of this gene in detail. In the present study, we describe the extent of CLPTM1L immunohistochemical expression in lung cancer tumor specimens, and we analyze the relationship between their expression and clinicopathological variables. We also conduct a trial to determine the possible function of this gene.

## Materials and Methods

### Patient’s Characteristics

Primary tumor specimens were obtained surgically from 151 lung cancer patients (110 men, age 39–81, median age at diagnosis 67 years; 41 women, age 36–85, median age at diagnosis 64 years) who had not undergone any preoperative therapy. Surgery was performed at the Shanghai Changning Central Hospital, Shanghai, China, between 2003 and 2010. These tumors included 55 cases of adenocarcinoma, 63 cases of squamous cell carcinoma, 13 cases of squamous-adenocarcinoma, 5 cases of small-cell lung cancer and 15 cases of large-cell lung cancer. The patients were staged according to the surgical and pathological findings based on the guidelines described in the *American Joint Committee on Cancer Staging Manual* [20]. Twenty-two patients were determined to be in stage Ia, 51 in stage Ib, 6 in stage IIa, 16 in stage IIb and 56 in stage IIIa. For all these patients, records of surgery, the in-patient medical records, chest x-ray films, whole-body computed tomography (CT) films and bone scanning films were reviewed. Paired normal tissues samples were taken to match specimens. The study was approved by the ethic committee of Putuo Hospital, Shanghai University of Traditional Chinese Medicine and all patients provided written informed consent.

### Cell Line and Maintenance

Human lung cancer cell lines 95-D was purchased from the Institute of Cell Biology (Shanghai, China) and maintained in RPMI 1640 medium supplemented with 10% FBS.

### CLPTM1L Expression Analysis Using Real-time Quantitative PCR

Total RNA was isolated from cultured cells using Trizol reagent (Invitrogen, Shanghai, China), according to the manufactuery’s protocol. First-strand cDNAs was prepared using random hexamer primer according to the instructions included with the SuperScriptIII™ first-strand synthesis kit (Invitrogen). Quantitative real-time PCR was performed using a Universal Master Mixer (Roche Applied Science, Shanghai, China) on a 7300 Real-time PCR System (Applied Biosystems, Shanghai, China). The primers and probes used were as follows: Forward, 5′- TGCATTACCTGCCCATCCT -3′; Reverse, 5′- CGCCCCAGTGAGACCTTG -3′; Probe, 5′-fam- TCATCGACCAGCTCAGCAACCGC -tamra-3′. GAPDH served as a control, F: 5′- CCACTCCTCCACCTTTGAC -3′, R: 5′- ACCCTGTTGCTGTAGCCA -3′, Probe: 5′-fam- TTGCCCTCAACGACCACTTTGTC -tamra-3′. Each assay was performed in triplicate. The PCR conditions used in all reactions were as follows: 10 min at 95°C, followed by 40 two-step cycles (95°C for 15 s and 60°C for 1 min). The relative expression levels of the CLPTM1L gene were normalized against GAPDH and analyzed by the 2^−ΔΔCt^ method [ΔΔCt = (Ct _CLPTM1L_ – Ct _GAPDH_ )sample - (Ct_ CLPTM1L_ – Ct _GAPDH_ )control].

### Cloning and Sequencing of Human CLPTM1L

Total RNA was isolated from 95-D cells using Trizol reagent (Invitrogen) and used as a template for first strand cDNA synthesis using a RT-PCR SuperScriptIII™ first-strand synthesis kit (Invitrogen). The CLPTM1L open frame (1617 bp, GenBank accession number NM_030782) was amplified by PCR from cDNA generated by reverse transcription of mRNA. The primers used to amplify the CLPTM1L gene were as follows: F: 5′- GGAAGATCTACCATGTGGAGCGGCCGCAG -3′; R: 5′- AGACGTCGACGTCCGTGTGGGGCGCC -3′. The amplified product was purified and cloned into a pMD18-T Simple Vector (Takara, Shanghai, China). The composition of the plasmid was confirmed by sequencing.

### Plasmid Construction and Gene Transfection

The CLPTM1L CDS region was extracted from pMD18-T- CLPTM1L plasmids by restriction digestion and cloned into a pEGFP-N3 vector. The constructed plasmid was named pEGFP-N3-CLPTM1L and it was used determine the cellular localization of CLPTM1L. Primers (F: 5′- GGAAGATCTACCATGTGGAGCGGCCGCAG -3′and R: 5′-CCGCTCGAGTCAGTCCGTGTGGGGCGCC-3′) were used to amplify the CLPTM1L CDS region for construction of pcDNA3.1(+) -CLPTM1L vector. The amplified product was purified and digested using Bgl II and Xho I. It was then cloned into pcDNA3.1(+) vector digested with BamH I and Xho I. The constructed plasmid was named pcDNA3.1(+) -CLPTM1L and used for CLPTM1L overexpression. 3 × 10^5^ 95-D cells were then transiently transfected with either pEGFP-N3-CLPTM1L or pEGFP-N3 by using Lipofectamine 2000 (Invitrogen, Shanghai, China) for 24 hr. Cells were then harvested for cell proliferation or cisplatin sensitivity analysis.

### Fluorescence Microscopy

To determine the subcellular localization of CLPTM1L-EGFP, the cells were first transiently transfected with pEGFP-N3-CLPTM1L using Lipofectamine 2000. Forty-eight hours after transfection, the cells were incubated with MitoTracker dye (Invitrogen) for 30 minutes under growth conditions. After incubation, cells were observed using a fluorescence microscope.

### Immunohistochemistry

Resected tissue specimens were fixed in formalin, embedded in paraffin, cut into 4 µm serial sections, and then mounted on glass slides. Antigen retrieval was performed using citrate buffer (0.01 mmol/L, pH 6. 0).After retrieval of the antigen, the slides were washed three times with PBS and incubated in 10% normal goat serum to block nonspecific background staining. Sections were then overnight incubated with rabbit anti-human CLPTM1L antibodies (Sigma, Shanghai, China) at 4°C. The sections were washed three times with PBS, and incubated with horseradish peroxidase (HRP)-anti-rabbit IgG (Maixin Bio, FuZhou, China) for 30 min. They were then washed three times with PBS. The sections were visualized using diaminobenzidine solution (DAB). Slides were evaluated simultaneously by two pathologists using a double-headed light microscope. The pathologists were not informed of each patient’s clinical record. CLPTM1L expression was semi-quantitatively scored based on the intensity of staining and relative number of cells stained. Unstained tissues was scored as 0, faint staining, moderate or strong staining in <25% of cells was scored as 1, moderate staining or strong staining in 25–50% of cells was scored as 2 and strong staining in >50% cells was scored as 3. Cell counts were performed at ×400 in at least five fields in randomly selected cancerous areas.

To study the relationship between CLPTM1L and the level of TIL infiltration, we selected the field with the largest amount of CLPTM1L expression, and counted the number of CD45^+^ cells per 1000 total nuclei.

### Immunocytochemistry

Expression of CLPTM1L protein was determined using immunocytochemistry. The day before the assay, a total of 1×10^5^ cells were seeded into Millicell EZ Slide (Millipore, Shanghai, China). After 24 h of incubation, cells were fixed on slides using 4% paraformaldehyde. The cells were permeabilized 3 times for 5 minutes with 0.1% Triton X-100 in PBS and blocked with blocking buffer (10% normal goat serum, 0.1% Triton X-100) for 30 minutes at room temperature. After blocking, the cells were washed with PBS and incubated overnight at 4°C with the rabbit anti-human CLPTM1L antibodies (Sigma, Shanghai, China). The following day, the cells were washed 3 times with PBS and incubated with horseradish peroxidase (HRP)-anti-rabbit IgG for 30 min. They were then washed three times with PBS. The sections were visualized using diaminobenzidine solution.

### Mitochondrial Purification and Western Bolt Analysis

First, 2×10^7^ 95-D cells were harvested and mitochondrial and cytosolic fractions were isolated using a Mitochondrial Fractionation Kit (Activemotif, Shanghai, China). The mitochondrial and cytosolic fractions were separated on a 10% SDS-PAGE, and then transferred to a PVDF membrane. The PVDF membrane was blocked with 5% BSA and washed twice with TBST. The membrane was then incubated with CLPTM1L antibodies (Abgent, Shanghai, China) overnight at 4°C, and washed three times with TBST followed by incubation with anti-rabbit IgG horseradish peroxidase secondary antibody (Cellsignal, Shanghai, China) for 2 h at room temperature. Finally, immunoreactive bands were detected using an ECL reagent (Millipore).

### Construction of CLPTM1L RNAi Lentivirus

siRNA sequences against the human CLPTM1L gene (GenBank accession number NM_030782) were designed and synthesized by Genechem (Genechem,Shanghai, China). Among four candidate siRNAs expression cassettes, we found sense siRNA sequence (5-CAGTTTCTGGAAGAAGAAGAA-3) to have the best interfering effect in our 95-D-infected cell system. The siRNA was inserted into pGCSIL-GFP lentiviral vector. A control siRNA (5-TTCTCCGAACGTGTCACGT-3) was used as a negative control. Lentiviruses encoded with siRNA against CLPTM1L and the control were produced by cotransfection of 293T cells using lipofectamine 2000 (Invitrogen) according to standard protocols. 5×10^4^ 95-D cells were infected with either CLPTM1L siRNA lentivirus or negative control siRNA vector at an MOI of approximately 100 for 72 hr. Cells were then switched into complete medium. After 72 hr culture, cells were harvested for cell proliferation, cisplatin sensitivity or caspase-3/7 and caspase-9 analysis.

### Cisplatin Sensitivity Analysis and Cell Proliferation Analysis

First, 1×10^4^ cells per-well were seeded into a 96-well plate. After 24 h of incubation, 25 µM, 50 µM and 75 µM cisplatin (Sigma) was added and incubated for 24 h. After 24 h, the live cell population was analyzed using Cell Proliferation Reagent WST-1 (Roche) according to manufacturer’s instructions. Cell proliferation was assessed at different points in time using WST-1.

### Analysis of Caspase-3/7 and Caspase-9 Analysis

The 95-D cells infected with CLPTM1L lentivirus and control lentivirus were cultured in RPMI 1640 medium supplemented with 10% FBS. Briefly, 1×10^4^ cells were seeded into a 96-well plate and incubated overnight. After 24 h, 95-D cells were treated with 50 µM cisplatin (Sigma) for 24 h. After 24 h, 100 µl of Caspase-Glo 3/7 or Caspase-Glo 9 reagent (Promega, Shanghai, China) was added to each well and incubated at room temperature for 2 h. After incubation, luminance was measured using TD 20/20 Luminometer (Promega). Each sample was measured in triplicate.

### Statistical Analysis

The associations between the expression of CLPTM1L and clinicopathological variables were analyzed using Fisher’s exact test, chi-square tests or continuity correction chi-square tests by SPSS16.0 software, and the relationships between the number of TIL and CLPTM1L were evaluated using the Mann-Whitney test using Instat3.36. For relationship between different markers, simple linear regression was performed using Statview SE+Graph.

## Results

### 1. Expression of CLPTM1L in Human Lung Cancer

**Figure 1:**
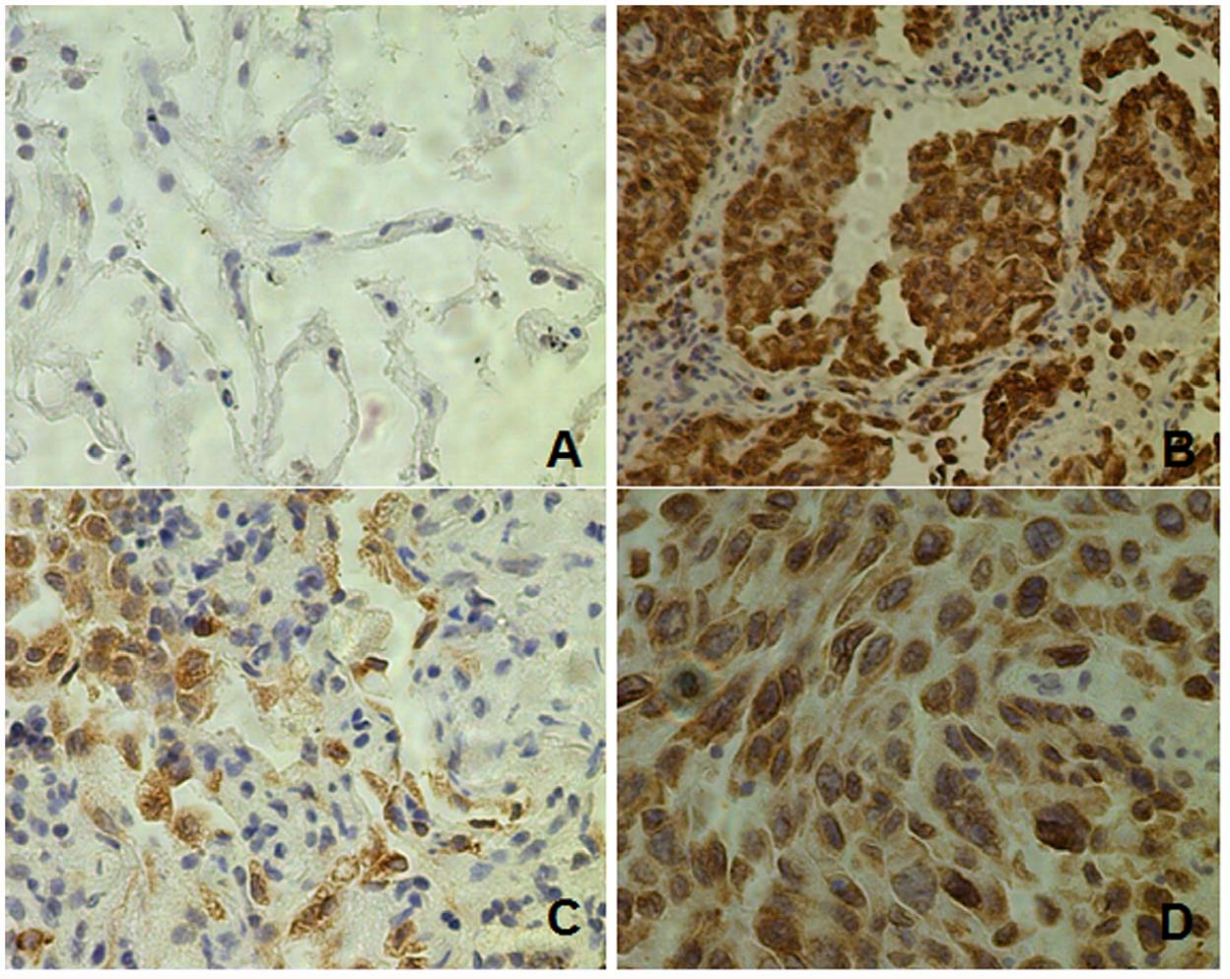
Representative images of immunohistochemical staining showing CLPTM1L is overexpressed in lung cancer relative to normal tissues. (A) Normal lung tissue (×200); (B) Section from lung adenocarcinoma (×200); (C) Section from lung squamous-cell carcinoma (×200); (D) Section from lung adenocarcinoma (×400).

The expression pattern of the CLPTM1L molecules on the surfaces of the tumor cells appeared very diffuse in most cases. Microscopically the CLPTM1L molecule was found in the cytoplasm (Fig. 1). In these specimens, infiltrating mast cells were found to express CLPTM1L strongly but lymphocytes were not. Out of the 151 patients who provided specimens, 136 (86.8%) showed CLPTM1L expression and CLPTM1L was overexpressed in tumor tissues compared to adjacent tissues, which was statistically significant different (p = 0.000, Table 1). The distribution of the intensity of staining in all specimens is shown in Table 1. A relationship between the intensity of staining and pathological classification was noted. The percentage of strong staining (++∼+++) of CLPTM1L expression in adenocarcinoma was higher than that in squamous-cell carcinoma (p = 0.000, Table 1). Because of the limited scale of the current study, the positive ratios of large-cell carcinoma and small-cell lung cancer could not be determined conclusively. The relative number of darkly stained cells was much higher in adenocarcinoma samples than in controls. Most adjacent tissues showed either weak staining or none at all.

**Table 1:** Immunochemistry of human lung tissue.

Tissue	Case	Staining intensity					P value
		−	+/−	+	++	+++	
tumors*	151	20	0	68	39	24	0.000
adjacent tissues	122	54	35	32	1	0	
Pathological type							
adenocarcinoma**	55	3	0	10	25	17	0.000
squamous	63	13	0	42	7	1	
adeno-squa mix	13	0	0	9	2	2	
small cell	5	2	0	1	0	2	
large cell	15	2	0	6	5	2	

* : p<0.01 vs. adjacent tissues,

** : p<0.01 vs. squamous.

### 2. Relationship between Clinicopathologic Characteristics and CLPTM1L Expression in Lung Cancer Patients

We found CLPTM1L expression was associated with the grades of differentiation (p = 0.046, Table 2), however no significant association was observed between CLPTM1L expression levels and patient age, sex, smoking status, or TMN stage in 151 lung samples (Table 2). No association was found between the expression of CLPTM1L and lymphocyte infiltration, which indicated that expression of CLPTM1L was not related to immunosuppression (Data not known).

**Table 2:** Clinical parameters.

Clinical parameter	patients No.	Expression CLPTM1L		
		negative	positive	P value
Age				
≤60	54	4	50	0.556
>60	97	10	87	
Sex				
Male	110	13	97	0.147
Female	41	1	40	
Smoking history				
P	98	9	89	1.000
N	53	5	48	
Differentiation				
Well*	59	2	57	0.046
not well	92	12	80	
Pathologic stage				
I	73	6	67	0.666
II+III	78	8	70	
pathologic T factor				
T1+T2	130	11	119	0.654
T3	21	3	18	
Pathologic N factor				
N0	73	6	67	0.666
N1+N2	78	8	70	

* : p<0.05 vs. not well differentiation group.

### 3. Mitochondrial Localization of CLPTM1L

**Figure 2:**
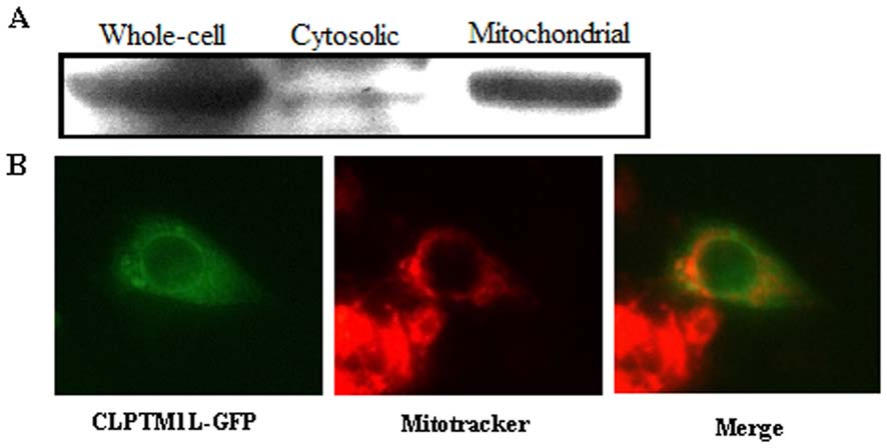
Cellular localization of CLPTM1L in 95-D cells. (A): Western blot analysis for CLPTM1L expression in whole-cell extract, cytosolic and mitochondrial fraction of 95-D cells. (B): 95-D cells were transiently transfected with CLPTM1L-EGFP vector and stained with the mitochondrial marker MitoTracker.

The exact cellular localization of CLPTM1L with respect to its roles in lung cancer progression is currently unclear. Examination of the sequence of CLPTM1L using MITOPROT (http://ihg2.helmholtz-muenchen.de/ihg/mitoprot.html) indicated an 84% probability that CLPTM1L was exported to the mitochondria. To determine the location of the CLPTM1L protein, mitochondrial and cytosolic fraction of 95-D cells were extracted for Western blot analysis. As shown in Fig. 2A, CLPTM1L protein was mainly found in the mitochondrial fraction of the cells. To confirm this, we constructed a CLPTM1L expression vector by fusing the EGFP at the C terminus of CLPTM1L (CLPTM1L-EGFP). Then 95-D cells were transiently transfected with CLPTM1L-EGFP vector and stained with the mitochondrial marker MitoTracker. The fluorescent images clearly indicated the mitochondrial localization of CLPTM1L protein (Fig. 2B).

### 4. Effect of CLPTM1L Overexpression in Infected 95-D cells

**Figure 3:**
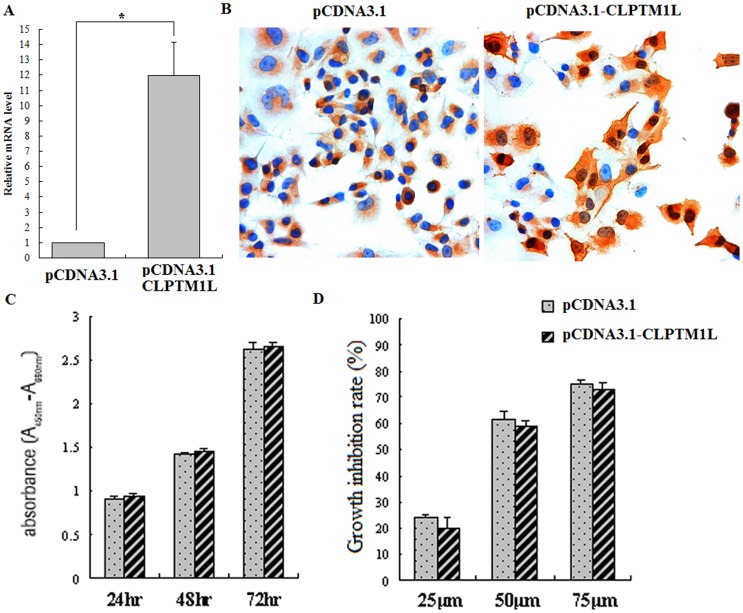
Overexpression of CLPTM1L in human lung cancer 95-D cells. (A) The CLPTM1L mRNA level was measured using quantitative real-time PCR in pcDNA3.1(+)-CLPTM1L or pcDNA3.1(+) plasmid transfected cells. *: *P*<0.05 vs control group (p = 0.001). (B) The expression of CLPTM1L protein was investigated using immunocytochemistry. (C) Effects of CLPTM1L overexpression on cell proliferation in the pcDNA3.1(+)-CLPTM1L transfected cells 95-D cells relative to control 95-D cells. (D) Overexpression of CLPTM1L did not change chemosensitivity to cisplatin in human lung cancer 95-D cells transfected with pcDNA3.1(+)-CLPTM1L relative to controls. The cells were treated with the indicated concentrations of cisplatin for 24 h.

To determine the functional consequences of elevated CLPTM1L expression in lung cancer, CLPTM1L was overexpressed in 95-D cells. Overexpression was confirmed using real-time PCR and immunocytochemistry (Fig. 3A and 3B). We did not find growth to be inhibited after 72 h of overexpression, as determined by proliferation analysis. Cells overexpressing CLPTM1L tended to be less likely to die when incubated with 25–75 µM cisplatin, though this difference was not found to be statistically significant (Fig. 3C and 3D).

### 5. Effects of CLPTM1L in Knocked Down 95-D Cells Infected by CLPTM1L shRNA Lentivirus

**Figure 4:**
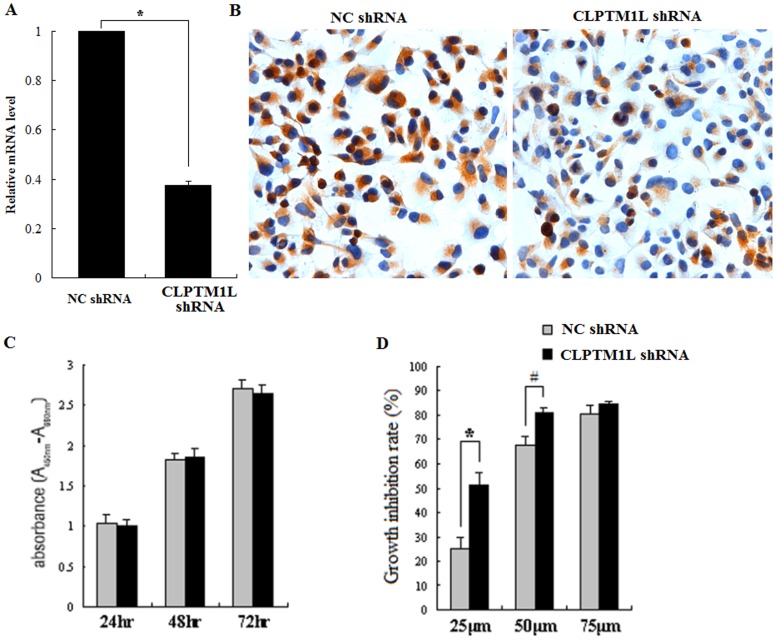
RNAi-mediated knockdown of CLPTM1L in human lung cancer 95-D cells. (A) The CLPTM1L mRNA level after RNAi treatment was measured using quantitative real-time PCR in different groups. *: p = 0.000 vs NC control group. (B) The expression of CLPTM1L protein after RNAi treatment was investigated using immunocytochemistry. (C) Growth of shRNA-CLPTM1L transfected cells 95-D cells and control 95-D cells for 72 h. (D) shRNA-CLPTM1L transfected cells 95-D cells and control 95-D cells were treated with indicated concentration of cisplatin for 24 h. *: p = 0.003 vs NC control group (25 µM) and #: p = 0.006 vs NC control group (50 µM).

We used a lentiviral vector containing shRNA to specifically target and stably knock down the expression of CLPTM1L in lung cancer 95-D cells. Real–time PCR analysis showed that CLPTM1L mRNA expression in shRNA-CLPTM1L-transfected cells was markedly lower than that of control 95-D cells (Fig. 4A). Decreased expression of CLPTM1L protein was also confirmed by immunocytochemistry (Fig. 4B).

To demonstrate the biological activity of CLPTM1L, we examined the effects of decreased CLPTM1L expression on lung cancer cell growth in vitro. Using a cell proliferation assay, we found that the shRNA-CLPTM1L-transfected 95-D cells had a growth rate similar to that of control cells over a 72 h period (Fig. 4C). CLPTM1L knockdown did not affect cell proliferation.

### 6. RNAi-mediated Knockdown of CLPTM1L Increased Chemosensitivity to Cisplatin in Human Lung Cancer 95-D Cells and Cisplatin-induced Activation of Caspase-9 and Caspase-3/7

CLPTM1L was first discovered in a cisplatin-resistant ovarian tumor cell line. Here we ascertained whether decreased CLPTM1L expression could increase chemosensitivity to cisplatin in lung cancer cells. Different concentrations of cisplatin were used to treat shRNA-CLPTM1L-transfected 95-D cells for 24 h. We found cell growth to be significantly inhibited in the knockdown cells after cisplatin treatment (Fig. 4D). This indicated that CLPTM1L knockdown could increase chemosensitivity to cisplatin in human lung cancer 95-D cells.

**Figure 5:**
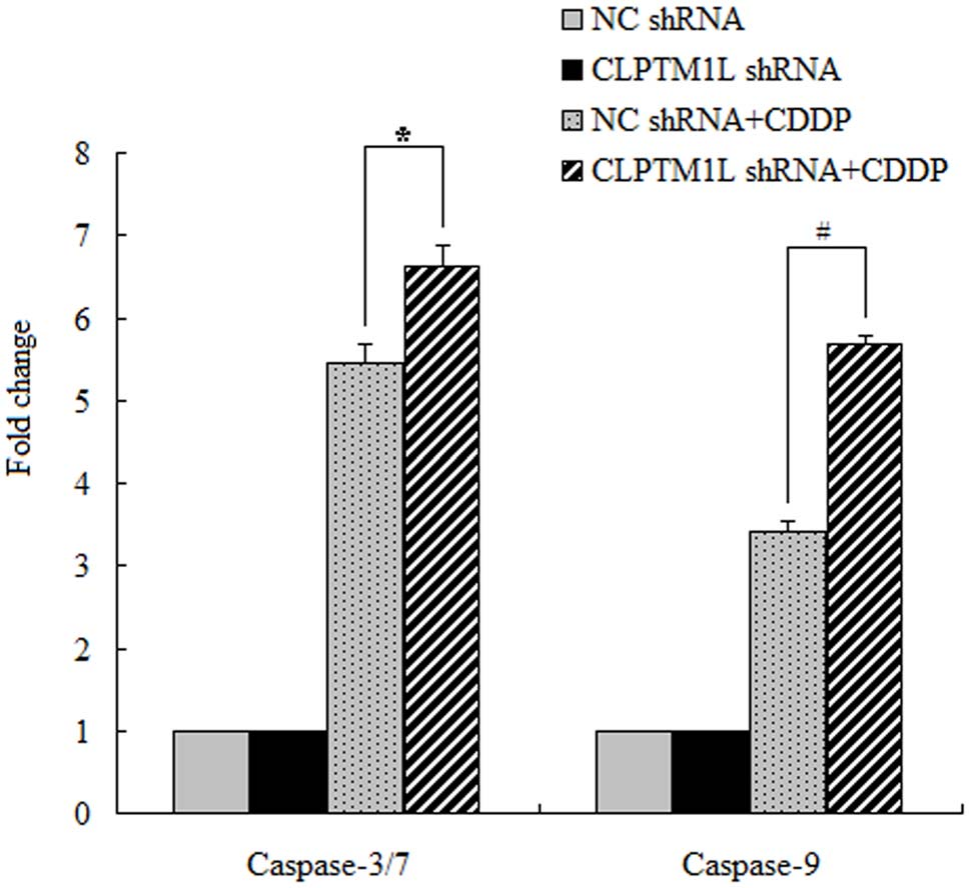
Knockdown of CLPTM1L increased cisplatin-induced activation of caspase-3/7 and caspase-9. shRNA-CLPTM1L transfected 95-D cells and control cells were treated with 50 uM cisplatin for 24 h. Caspase-3/7 and caspase-9 activity were measured and the results were represented as fold increase of the activity of the cells without cisplatin treatment. *: p = 0.004 vs NC control group (caspase-3/7) and #: p = 0.000 vs NC control group (caspase-9).

Mitochondria are key to the regulation of apoptosis [21], and they play an important role in cisplatin-induced apoptosis. After treated with cisplatin, caspase-9 and caspase-3/7 in mitochondria were activated. Since CLPTM1L protein was found to be exported to the mitochondria, we wondered if CLPTM1L was associated with apoptostic protein. We then examined the activation of caspase-9 and caspase-3/7 in shRNA-CLPTM1L-transfected 95-D cells and control 95-D cells to determine whether CLPTM1L was involved in the mitochondrial apoptosis pathway. Our results showed that cisplatin-induced apoptosis to begin with the activation of caspase-9, followed by the activation of caspase-3/7 in both cell types. Furthermore knockdown of CLPTM1L caused increased activation of caspase-9 and caspase-3/7 (Fig. 5). This means that CLPTM1L knockdown cells are more sensitive to cisplatin.

## Discussion

In the hope of defining the pathogenesis of CLPTM1L in lung cancer, we focused on CLPTM1L expression, cellular localization and functional association with lung cancer. We found that CLPTM1L was expressed in cancerous lung tissue, most intensely in adenocarcinoma tissue. Western-blot analysis and CLPTM1L-EGFP transfection both indicated that the molecule may be located in the mitochondria. The exact function of the molecule is still unclear. We ascertained its association with chemosensitivity to cisplatin and activation of the mitochondrial apoptotic pathway depending on RNAi knock-down technique. To the best of our knowledge, this is the first evidence of location of the molecule.

This study provided more evidence that CLPTM1L was associated with lung cancer [22], which was consistent with the published study by James et al and The Human Protein Atlas project [23]. James et al investigated the expression of CLPTM1L in mRNA level and found CLPTM1L mRNA expression was an average of 2.24 fold higher in tumor tissues compared to tumor-adjacent tissues [23]. When combined with James’ work, our observation appeared to indicate that the elevated CLPTM1L protein levels might resulted from the increase in CLPTM1L mRNA levels. In addition, we compared relationship between CLPTM1L expression in lung cancer patients with patients’ clinicopathologic characteristics. We found CLPTM1L expression was strongly associated with the grades of differentiation (p = 0.046, Table 2), however, no significant association was observed between CLPTM1L expression levels and patient age, sex, smoking status, or TMN stage in 151 lung samples. Furthermore, the percentage of strong staining of CLPTM1L expression in adenocarcinoma was higher than that in squamous-cell carcinoma, although James et al did not find a difference in CLPTM1L expression between NSCLC subtypes. The difference between our results and James’ results might be caused by the number of patients. James analyzed 22 adenocarcinoma and 8 squamous cell carcinoma patients while we analyzed 55 adenocarcinoma and 63 squamous cell carcinoma patients.

Here we confirmed that the CLPTM1L protein was more highly expressed in lung cancer, especially in adenocarcinoma, than in normal tissue, This indicated that the practical application of the genetic variations of the gene was not limited to use as genetic markers. The incidence of lung adenocarcinoma has been increasing markedly. The etiology is commonly believed to involve air pollution and secondhand smoke. However, carcinogenesis [19], [24] is complicated, and the CLPTM1L gene may also play an important role. Genetic analysis has revealed that CLPTM1L is closely associated with lung adenocarcinoma and the genetic variance may cause abnormal gene expression, which plays an important role in the pathogenesis of lung cancer.

The protein seems to be localized in mitochondria, as indicated by Western-blot and CLPTM1L-EGFP transfection. This conclusion is supported by protein prediction. The calculation of hydrophilicity indicates that CLPTM1L protein might be a transmembrane protein. According to these data, we asserted that this protein was a transmembrane protein located on mitochondria membrane.

The exact function of CLPTM1L is still unknown. James et al indicated that CLPTM1L had an apoptotic role downstream of DNA damage and through regulation of Bcl-xL expression [23]. In this study, we could not determine the exact biologic changes associated with CLPTM1L overexpression and knock-down in lung cancer cell lines. In contrast to the result of previous report [1], overexpression of CLPTM1L in 95-D lung cell line did not induce apoptosis and the cells tends to be less chemosensitive to cisplatin [1]. The difference may have been caused by expression vectors, which may have induced different products: CLPTM1L-His for the present study and CLPTM1L-GFP for previous study. RNAi-mediated knockdown of CLPTM1L increased chemosensitivity to cisplatin in human lung cancer 95-D cells and cisplatin-induced activation of caspase-9 and caspase-3/7. Increased activation of caspase-9 and caspase-3/7 might be associated with increased activation of the mitochondrial apoptosis pathway. We still found that the mast cells highly expressed the protein. It is commonly accepted that mast cells are associated with cancer, they enhance tumor angiogenesis and render progress less favorable [25]. According to these data, we predicted that the protein might be associated with anti-apoptotic mechanism which affected drug-resistance. Further analysis is necessary to confirm this prediction.

The specific roles of CLPTM1L in lung cancer cells merit further investigation. It is possible that CLPTM1L may be important to the maintenance of cellular stability and that a loss of function might result in increased chemosensitivity to cisplatin.
